# 'Experience talks': physician prioritisation of contrasting interventions to optimise management of acute cough in general practice

**DOI:** 10.1186/1748-5908-4-57

**Published:** 2009-09-08

**Authors:** Jochen WL Cals, Christopher C Butler, Geert-Jan Dinant

**Affiliations:** 1 Department of General Practice, School for Public Health and Primary Care (CAPHRI), Maastricht University Medical Centre, The Netherlands; 2 Department of primary care and public health, School of Medicine, Cardiff University, Cardiff, UK

## Abstract

**Background:**

Uptake of interventions to improve quality of care by clinicians is variable and is influenced by clinicians' attitudes. The influence of clinicians' experience with an intervention on their preference for adopting interventions is largely unknown.

**Methods:**

Thematic analysis of semi-structured interviews exploring views and attitudes towards an illness-focused intervention (specific communication skills training) and a disease-focused intervention (C-reactive protein, or CRP, point-of-care testing) to optimize management of lower respiratory tract infections (LRTI) among general practitioners (GPs) who had used both interventions for two years in a randomised trial (exposed GPs), and GPs without experience of either intervention (non-exposed GPs).

**Results:**

All but two of the ten non-exposed GPs indicated that they would prioritise implementation of the disease-focused intervention of CRP testing over communication skills training, while all but one GP in the exposed group said that they would prioritise the illness-focused approach of communication skills training as it was more widely applicable, whereas CRP testing was confirmatory and useful in a subgroups of patients.

**Conclusion:**

There are differences in attitudes to prioritising contrasting interventions for optimising LRTI management among GPs with and without experience of using the interventions, although GPs in both groups recognised the importance of both approaches to optimise management of acute cough. GPs' experiences with and attitudes towards interventions need to be taken into account when planning rollout of interventions aimed at changing clinical practice.

## Introduction

Achieving effective uptake of new evidence into routine clinical care is challenging. Several barriers and enablers to evidence uptake have been identified. These range from practice environment and organisational factors to professional knowledge and attitudes [1].

Continuing professional development is concerned with the acquisition, enhancement, and maintenance of knowledge, skills, and attitudes. Learning and improving practice, including uptake of new interventions, is mainly governed by individual clinicians' motivation and perceived needs [2]. However, clinicians may not choose to adopt the most effective or important interventions and identifying factors influencing health professionals' behaviours is challenging [3].

For example, contrasting approaches have been suggested for enhancing physician antibiotic prescribing practices [4]. A disease-focused perspective promotes interventions to decrease diagnostic uncertainty such as diagnostic tests. An illness-focused perspective promotes interventions aimed at addressing the patients' agenda, such as physician communication skills training. However, it is not known how experience with one or other of these broad approaches influences GPs perceptions about which intervention type they would prioritise for adoption into their own practice.

We therefore studied the role of experience with interventions in influencing clinician prioritising of intervention uptake. We focused on two contrasting interventions for improved management of the exemplar condition of lower respiratory tract infections (LRTI) in general practice [5]. We describe the attitude of physicians with experiences of implementing both approaches and the attitude of physicians who have no practical experience of either intervention. Our goal was to highlight the influence of physician exposure to contrasting approaches when considering prioritising interventions for adoption into their clinical practice.

## Methods

### IMPAC^3^T trial

We analysed qualitative interview data obtained from general practitioners (GP) who participated in the IMPAC^3^T trial (Improving Management of Patients with Acute Cough by C-reactive protein testing and Communication skills Training, ISRCTN85154857) [5]. This study was a factorial, cluster randomised clinical trial assessing the effect of two contrasting interventions, singly and combined, on antibiotic prescribing for LRTI. These interventions were:

1. Disease-focused: C-reactive protein (CRP) point-of-care testing, assisting GPs to differentiate serious from self-limiting LRTI.

2. Illness-focused: Clinician communication skills training, assisting GPs to provide evidence-based information on the natural course of LRTI and setting realistic expectations on the role of antibiotics for LRTI.

The trial protocol [5] and description of the clinician communication skills training [6] as well as the effectiveness [7] and cost-effectiveness (Cals JWL, Ament AA, Hood K, Butler CC, Hopstaken RM, Wassink GF, Dinant G: Cost-effectiveness of C-reactive protein point of care testing and physician communication skills training in reducing antibiotic prescribing for lower respiratory tract infections in general practice, submitted) of the two interventions have been described elsewhere. In brief, both interventions were effective at reducing antibiotic prescribing for LRTI, without compromising clinical outcome or patient satisfaction. Both interventions were cost-effective from the health care perspective. As part of the process evaluation, we interviewed all participating GPs after trial completions to explore their experiences with and attitudes towards the interventions.

Because GP practices were randomised in the trial, we had the unique opportunity to explore views and attitudes towards these contrasting interventions of two distinct groups of GPs:

1. GPs exposed to both interventions: 10 GPs had used both interventions (CRP and communication skills) for at least two years (exposed GPs)

2. GPs not exposed to either intervention: 10 GPs practicing as usual without either of the two interventions during these two years (non-exposed GPs)

### Interview procedure

We used qualitative research methods as these are best suited to achieving a deep understanding of experiences and views from the perspective of the physicians (rather than quantifying the pre-conceived notions of researchers) [8]. We conducted individual, semi-structured interviews in GP surgeries. The average length was 30 minutes. Interviews were audio taped and took place in the first winter after the end of the trial. The GPs were told that our purpose was not to audit or pass judgement on practice but to understand their experiences and views. At the time of the interview, GPs were unaware of the trial results. Two trained interviewers conducted semi-structured interviews. For unexposed GPs, we extensively described the interventions and asked them about the possible impact on their own practice and about their preferences for prioritising the interventions. The interview guide was piloted in one videotaped interview. All questions were open, followed by predetermined prompts when there was no response to the initial question. We aimed to interview all 20 study GPs. The main question in the interview schedule that generated data for the present analysis was: Which intervention would help you most improving your management of LRTI and why?

### Data analysis

The audiotaped interviews were transcribed by an experienced medical typist. Three researchers then read the transcripts. Analysis and data collection were conducted in parallel. Coding schedules were agreed upon and piloted. Seventy percent of the interviews were double coded, with the remaining transcripts coded by only one researcher. Discrepancies were resolved by discussion whenever possible. Where disagreement remained, a third researcher (JC) was consulted who made the final decision. We sought to identify commonly expressed themes as well as unusual cases using thematic content analysis. This method of analysis is essentially a process of summarization, categorization, and counting frequency of responses [9]. Data analysis and reporting was assisted by NVivo software.

## Results

### Prioritising the illness or disease-focused intervention

All 20 GPs in the relevant randomisation groups in the cluster randomised controlled trial agreed to be interviewed. GPs' characteristics in each group were similar and comparable to average Dutch GPs [7]. The quotations in Table 1 illustrate that GPs in the two groups expressed contrasting initial reactions in answer to the key question (Which intervention would help you most improving your management of LRTI and why?)

**Table 1:** Preferences of exposed and non-exposed GPs towards illness or disease-focused interventions to improve LRTI management

**Exposed GPs**	**Preference**
'You can't take them apart, I want them both.' (GP1)	Undecided
'I found CRP less useful than communication skills training.' (GP2)	CST
'Communication is the key component of our profession. If not doing it [communication skills as thought in the training] yet, one should immediately consider it. The other thing [CRP] is an addition, but a very useful one in my opinion.' (GP7)	CST
'Communication, as I think this is the most important in consultations, either in LRTI or another condition... CRP as a value is wonderful, but it doesn't tell you everything.' (GP8)	CST
'Communication skills training.' (GP9)	CST
'If I really need to choose I need to say communication skills training.... That was great fun to do, to systematically use it, it works.' (GP10)	CST
'The communicative bit has my preference yes. I always try to do without the test, but well, if I don't succeed I pull out CRP to convince patients.' (GP13)	CST
'Communication. I try to structure my consultation to give attention to all aspects, and CRP can be one of them.' (GP14)	CST
'Communication training... in the majority of patients you do well with these skills, and when in doubt with CRP.' (GP17)	CST
'You'll achieve most by doing communication skills training.' (GP18)	CST

**Non-exposed GPs**	**Preference**

'I'd choose CRP. Two reasons: I'm a games person, so I love such a test very much... and because I feel that communication skills training thing, well... I don't think I need to improve that much in that field... I don't feel communication is the problem in antibiotic prescribing.' (GP3)	CRP
'Yes, CRP.' (GP4)	CRP
'CRP, as it is useful in my practice and because I feel I can get patients on my side with it. I think that the magic of the machine is more than the magic of my words.' (GP5)	CRP
'Most obvious would be CRP, easier and less time consuming.' (GP6)	CRP
'CRP, as I think that I will not improve that much when knowing how to give information back to the patient, but I would find it useful to have such a test in my practice.' (GP11)	CRP
'I think communication training, as I can buy CRP myself.' (GP12)	CST
'CRP, it is useful to know that it is there to be used.' (GP15)	CRP
'CRP could help me in case of doubt, I don't see how communication would help me in that regard.' (GP16)	CRP
'In the end communication skills training will benefit the most. It is, in any form, always an eye-opener and even if only small bits are remembered, it is nice.' (GP19)	CST
'CRP for sure. Much easier, much faster. I expect more of it than of communication skills training. Such a training will offer some extra, but not much.' (GP20)	CRP

CST = Communication skills training (Illness focused approach)

CRP = C-reactive protein point-of-care testing (Disease focused approach)

All but two of non-exposed GPs indicated they would prefer to adopt the disease-focused intervention of CRP testing to optimise management of LRTI in their practice. This contrasted with the exposed GPs, where all but one indicated they favoured the illness-focused approach of enhanced communication skills training for LRTI management. The one exception in this group declined to make a choice, as he felt both approaches should always be integrated.

### Non-exposed GPs choosing the disease-focused intervention

Non-exposed GPs expressed favourable attitudes to CRP point-of-care testing relating to the professional context of their working environment. 'I'm convinced that it will enhance diagnostic certainty' (GP5, non-exposed), and 'I'm sure that half of all prescriptions are not necessary and CRP is useful for confirming this assumption before actually making the decision about prescribing' (GP19, non-exposed). These attitudes arise from a disease-focused concern to rule out serious disease. Yet, achieving shared decisions with patients not to prescribe antibiotics was also frequently mentioned by nearly all non-exposed GPs. 'CRP would be useful in my practice and because I feel I can get patients on my side with it. I think that the magic of the machine is more powerful than the magic of my words' (GP5, non-exposed).

This last quote is typical of non-exposed GPs who attached greater value to CRP testing compared to enhanced communication skills training. A typical quote from a non-exposed GP addressed barriers: 'I don't think that this is where my weakness is' (GP3, non-exposed), and 'it [communication skills training] is never real life, training only tells you how you could or may do it' (GP16, non-exposed). Four non-exposed GPs stated that communication skills training was not a priority for them, and four others said they already deployed excellent communication skills. Non-exposed GPs were sceptical of the value of the time investment required for enhanced communication skills training. They were also concerned by the potential negative impact on consultation length of focusing on communication about antibiotics with their patients. So, many non-exposed GPs did not feel any compulsion to act in this regard. 'What we want as GPs must fit in 10-minute consultations. So as long as we aim to do these things [communication skills] within the time restriction of 10 minutes, implementing communication skills will not be feasible' (GP5, non-exposed).

Despite the overwhelming preference for CRP testing as their priority intervention, all but one of the non-exposed GPs also expressed positive attitudes towards illness-focused communication in LRTI. Typical comments were: 'In the end it [good communication] will lead, so we hope, to a satisfied patient, a satisfied GP, and less antibiotic use' (GP12, non-exposed), and 'I always do it, I find it the most important part of my professional practice' (GP15, non-exposed).

### Exposed GPs choosing the illness-focused intervention

All but one of the GPs exposed to both interventions indicated that if they had to choose, they would select the illness-focused intervention over the disease-focused intervention. The remaining GP preferred not to make a choice as he felt both approaches should always be integrated. However, all exposed GPs also saw a place for CRP testing as for some, but not all, patients with LRTI. Typical quotes are: 'I think these communication skills are more essential, with CRP giving additional guidance' (GP10, exposed), and 'communication is of utmost importance in general practice. More important even than drugs, so I find this communication skill training crucial and CRP is a useful addition' (GP18, exposed). Eight GPs indicated that they used their enhanced communication skills with all patients and used CRP only when faced with particular problems: 'It depends on the patient. For some patients, [CRP] could be of additional value, but some I think will do fine without the test and the communication bit is more than adequate, while some patients want more objective measures [like CRP]. It certainly depends on the patient which strategy I choose' (GP13, exposed).

### The best of both worlds?

Despite differences in prioritising the interventions, both groups acknowledged a central role for both approaches to optimise management of acute cough, albeit from different perspectives.

In general, exposed GPs stressed the value of having both approaches. One-half suggested the interventions would be synergistic, and all agreed that having the combination available would be ideal. 'I think you can combine both quite nicely, it is additive, like I said before. It is a very natural combination, very complete indeed' (GP7, exposed). 'Management decisions are more robust if you combine them' (GP9, exposed), and 'CRP is a confirmation of your account of things. If they [patients] hear that their blood test was also normal, your explanation becomes even more credible to patients' (GP2, exposed). Although the dominant view was that good communication skills would be adequate for optimal handling of most consultations, the GP's own agenda, including dealing with diagnostic uncertainty, was not forgotten and here CRP testing had a role: 'You can use it when patients are in doubt [not convinced], but certainly also when you yourself are uncertain' (GP17, exposed). Time constraint was the only commonly mentioned disadvantage of utilising both approaches within LRTI consultations. However, GPs in this group did not see this as a barrier to implementing the approaches. 'It takes a bit more time, but I do think that we then confirm the decisions from two angles, which provides more satisfaction and reassurance' (GP2, exposed).

Non-exposed GPs recognised the value of both approaches but were nevertheless inclined to express a preference for the CRP approach over the other. On the one hand, to decrease diagnostic uncertainty, but also to convince patients: 'CRP would be useful in my practice and because I feel I can get patients on my side with it. I think that the magic of the machine is more powerful than the magic of my words' (GP5, non-exposed). However, they saw good communication skills as a key competence for daily practice anyway, for example: 'I have been [a] GP for a long time and this is something I have always been mindful of, structured and focused communication. That's always something I strive for, time and time again, and I'll keep doing it until you get sick of it' (GP15, non-exposed).

## Discussion

This study found differences in GPs' expressed preferences for prioritising contrasting interventions to optimise LRTI management. Those GPs who had experience of both an illness-focused intervention (communication skills training) and a disease-focused intervention (CRP point-of-care test) indicated that they would choose to prioritise enhanced communication skills. Conversely, GPs without access to CRP point-of-care testing and enhanced communication skills training indicated they would prefer to have access to the CRP disease-focused intervention.

The views and attitudes expressed in this study must be considered in the context of the quantitative findings from the randomised factorial trial [7]. Here, our primary analysis considered an issue of discrete choice about which intervention GPs would prioritise. Apart from the striking differences between the exposed and non-exposed clinicians in relation to the study question, many similarities between the two groups were identified. Both recognised a place for both approaches in the management of acute cough.

These findings may be helpful when considering barriers to, and incentives for achieving evidence-based practice and implementation, a process which is receiving greater research and policy attention [10]. Although non-exposed GPs saw skilled communication as a core competency for daily practice, our data did not indicate a hunger for improving specific communication skills to better manage LRTI. Such professional barriers will determine whether or not an intervention is successfully adopted into routine care. On the other hand, GPs who had been exposed to the interventions saw a role for enhanced communication skills in all LRTI consultations. They stated that the CRP disease-focused intervention could be useful in managing a subgroup of patients.

This study included selected GPs -- those that had recently participated in a RCT. Their views may no be typical of GPs' views on prescribing decisions and antimicrobial resistance [11-15]. GPs' accounts of their experiences of CRP point-of-care testing for LRTI in this trial have been reported elsewhere (Cals JWL, Chappin FHF, Hopstaken RM, van Leeuwen ME, Hood K, Butler CC, Dinant GJ: C-reactive protein point of care testing for lower respiratory tract infections; a qualitative evaluation of experiences in general practice, submitted). While non-exposed GPs did not have access to the interventions, they had been recruiting LRTI patients into the trial over two winters, and some contamination may have occurred. We did not explore patients' views in this research. However, we do know from the trial data that participating patients were highly satisfied with their consultations, irrespective of the intervention their managing clinician was exposed to during the study [7].

Exposed GPs recognised that effective communication is the foundation of good medical practice. They also recognised the importance of the enhanced communication skills intervention for optimising the management of a specific condition, LRTI. Nevertheless, they did indicate that differentiating serious from self-limiting disease is a crucial component of their professional role. They found CRP testing valuable in a specific subgroup of patients, namely those who were not convinced of management decisions based on history and physical examination alone. It would be erroneous to conclude that exposed GPs would only want to use their communication skills and never use CRP point-of-care testing. All exposed GPs indicated that CRP testing had a useful role in LRTI management. Similarly, non-exposed GPs recognised the value of communication skills training in general, although they considered that they would find CRP point-of-care testing more useful. This may also be explained by how enhanced communication skills and a diagnostic test were conceptualised by this non-exposed group. Because communication is seen as already essential to good medical care, an intervention to further expand these skills may be seen as less important than a new diagnostic test, which adds to the physician's agenda of increasing diagnostic certainty. Similarly, a test result can also be seen as an aid to persuade patients to accept certain management decisions, exemplified by a striking quote by non-exposed GP5 (see Table 1). Both interventions can affect the communication dynamics within a consultation and, despite the fact that a diagnostic test is a disease-focused intervention, it may affect the illness experience of the patient as well.

As with previous research, GPs in this study were concerned about the impact of using enhanced communication skills and shared decision making on consultation length [16,17]. Implementing communication skills did not increase consultation time beyond feasible limits during competence assessment [6]. Nonetheless, exposed GPs recognised that extra time invested in combining both approaches would be synergistic, providing enhanced reassurance from two directions.

Setting priorities for uptake of contrasting interventions may differ substantially between GPs with and without previous exposure to the interventions. GPs' level of experience with and attitudes towards interventions to improve clinical practice need to be taken into account when planning widespread dissemination.

## Competing interests

The authors declare that they have no competing interests.

## Authors' contributions

JC is the principal investigator and wrote the manuscript. All authors have read and approved the final version of the manuscript
